# Shotgun-Metagenomics on Positive Blood Culture Bottles Inoculated With Prosthetic Joint Tissue: A Proof of Concept Study

**DOI:** 10.3389/fmicb.2020.01687

**Published:** 2020-07-17

**Authors:** Adriana Sanabria, Erik Hjerde, Mona Johannessen, Johanna Ericson Sollid, Gunnar Skov Simonsen, Anne-Merethe Hanssen

**Affiliations:** ^1^Research Group for Host-Microbe Interactions, Department of Medical Biology, Faculty of Health Sciences, UiT – The Arctic University of Norway, Tromsø, Norway; ^2^Department of Chemistry, Centre for Bioinformatics, UiT – The Arctic University of Norway, Tromsø, Norway; ^3^Department of Microbiology and Infection Control, University Hospital of North Norway, Tromsø, Norway

**Keywords:** shotgun-metagenomics, clinical metagenomics, prosthetic joint infection, blood culture bottles, prosthetic joint tissue

## Abstract

Clinical metagenomics is actively moving from research to clinical laboratories. It has the potential to change the microbial diagnosis of infectious diseases, especially when detection and identification of pathogens can be challenging, such as in prosthetic joint infection (PJI). The application of metagenomic sequencing to periprosthetic joint tissue (PJT) specimens is often challenged by low bacterial load in addition to high level of inhibitor and contaminant host DNA, limiting pathogen recovery. Shotgun-metagenomics (SMg) performed directly on positive blood culture bottles (BCBs) inoculated with PJT may be a convenient approach to overcome these obstacles. The aim was to test if it is possible to perform SMg on PJT inoculated into BCBs for pathogen identification in PJI diagnosis. Our study was conducted as a laboratory method development. For this purpose, spiked samples (positive controls), negative control and clinical tissue samples (positive BCBs) were included to get a comprehensive overview. We developed a method for preparation of bacterial DNA directly from PJT inoculated in BCBs. Samples were processed using MolYsis5 kit for removal of human DNA and DNA extracted with BiOstic kit. High DNA quantity/quality was obtained, and no inhibition was observed during the library preparation, allowing further sequencing process. DNA sequencing reads obtained from the BCBs, presented a low proportion of human reads (<1%) improving the sensitivity of bacterial detection. We detected a 19-fold increase in the number of reads mapping to human in a sample untreated with MolYsis5. Taxonomic classification of clinical samples identified a median of 96.08% (IQR, 93.85–97.07%; range 85.7–98.6%) bacterial reads. Shotgun-metagenomics results were consistent with the results from a conventional BCB culture method, validating our approach. Overall, we demonstrated a proof of concept that it is possible to perform SMg directly on BCBs inoculated with PJT, with potential of pathogen identification in PJI diagnosis. We consider this a first step in research efforts needed to face the challenges presented in PJI diagnoses.

## Introduction

Metagenomic next-generation sequencing (mNGS) refers to shotgun sequencing of all available DNA and/or RNA in a sample followed by the precise taxonomic identification and classification of each sequence (Couto et al., 2018; Rutanga et al., 2018). The application of shotgun metagenomics (SMg) to clinical samples to recover information of clinical relevance is emerging and it is known as clinical metagenomics (Forbes et al., 2018).

Shotgun-metagenomics has a huge potential, particularly in areas where conventional diagnostic methods have limitations such as in prosthetic joint infection (PJI) (Thoendel et al., 2018). It is a promising approach opening huge opportunities for detecting, identifying and characterizing all potential pathogens, providing at the same time additional inputs on important characteristics for clinical management, such as antibiotic resistance determinants, virulence factors, and bacterial evolution (Wilson et al., 2019).

To date, several studies have provided evidence of the potential and successful applications of clinical metagenomics in a variety of clinical specimens including urine (Hasman et al., 2014; Schmidt et al., 2017), respiratory secretions (Nakamura et al., 2009; Bogaert et al., 2011; Schlaberg et al., 2017; Langelier et al., 2018), cerebrospinal fluid (Schlaberg et al., 2017; Miller et al., 2019), stool (Zhou et al., 2016), blood (whole blood, serum, and plasma), and tissue (Ruppé et al., 2017). Several reviews have summarized the advances, limitations and challenges in the field (Padmanabhan et al., 2013; Simner et al., 2018; Chiu and Miller, 2019).

There are many issues that need to be addressed before performing clinical metagenomics in real time directly from clinical samples as an integral part of routine diagnostic testing. Among them are the technical challenges related to sample preparation protocols, to process and analyze clinical specimens. In this regard, it is important to consider that different specimen types present their unique and specific challenges reflecting their matrix and concentrations of the target pathogen(s) and normal (resident) flora (Bachmann et al., 2018). Therefore, when performing sequencing directly from a clinical sample, special attention has to be paid to (1) contamination of host DNA and other microorganisms, (2) low abundance of the target organism present in the sample and (3) the presence of DNA amplification inhibitors and other sample variables (i.e., sample type, matrix) (Mulcahy-O’Grady and Workentine, 2016; Bachmann et al., 2018).

Prosthetic joint infection (PJI) is a serious complication after joint implantation, occurring in 1–2% of primary arthroplasties and 4% in revision arthroplasties (Ong et al., 2009; Corvec et al., 2012; Izakovicova et al., 2019). The infection is associated with high morbidity rates and diagnosis is challenging due to imperfect definition and imperfect diagnostic techniques (Tande and Patel, 2014; Park and Patel, 2018). Undiagnosed PJI cases due to culture-negatives have been estimated to a proportion of 10–30%, for example due to ongoing empirical antibiotic treatment (Tande and Patel, 2014; Peel et al., 2016). Culturing the causative pathogen takes longer time and is problematic in less virulent, fastidious and slow growing organisms (Schafer et al., 2008).

The diagnosis of PJI is not standardized (Parvizi et al., 2011). However, the microbiological assessment of periprosthetic tissue is the most important method and to date the gold standard for diagnosing PJI (Parvizi et al., 2011; Yan et al., 2018). Due to low sensitivity and specificity, rapid and accurate diagnosis is still a challenge. Additionally, methods for rapid pathogen identification directly on clinical samples such as multiplex PCR, MALDI-TOF and whole genome sequencing (WGS) have been developed (Greenwood-Quaintance et al., 2014; Patel, 2015; Tagini and Greub, 2017). However, they are still dependent on pure microbial culture in addition to the fact that some of them do not give information beyond species identification (Török et al., 2013). Research and development of new diagnostic methods that overcome these limitations are required.

In a previous study, we assessed the use of a BacT/Alert^®^ Virtuo blood culture system for culturing periprosthetic tissue specimens (Sanabria et al., 2019). The blood culture bottle (BCB) method was found to detect a wider range of bacteria more rapidly than the conventional microbiological method. Furthermore, previous studies have demonstrated the potential of microbiological detection using BCBs for culturing specimens related to PJI, such as synovial fluid (Hughes et al., 2001; Font-Vizcarra et al., 2010), sonication fluid (Portillo et al., 2015; Shen et al., 2015; Janz et al., 2016; Dudareva et al., 2018), and prosthetic joint tissue (PJT) (Velay et al., 2010; Minassian et al., 2014; Peel et al., 2016, 2017; Dudareva et al., 2018). The evaluation of SMg in the diagnosis of PJI remains limited (Zhang et al., 2019). To date, studies have investigated the application of SMg on tissue specimens (Ruppé et al., 2017), synovial fluid (Ivy et al., 2018) and sonication fluid (Thoendel et al., 2016, 2017, 2018; Street et al., 2017; Sanderson et al., 2018; Yan et al., 2019), where the main obstacle has been a high background of genetic material mainly derived from the host, hampering the detection of pathogens (Zhang et al., 2019).

So far, there are no studies about SMg on BCBs inoculated with any specimens related to PJI. We believe that the combination of inoculation of BCBs with PJT specimens followed by direct DNA sequencing may be a beneficial strategy. Here, we developed a proof of concept study with the aim of evaluating the use of SMg on BCBs inoculated with PJT for pathogen identification in diagnosis of PJI. Our study was conducted as a laboratory method development, including PJT specimens and appropriate controls. The aim was to test if SMg technology works on this specific type of specimens, and for this purpose develop a method for preparation of high-quality bacterial DNA from PJT for downstream SMg, establish a bioinformatics pipeline, and compare SMg results with conventional culture method results. SMg was performed to investigate if it was possible to obtain an acceptable high number of bacterial reads, genome coverage and genome sequencing depth for identification of PJI pathogens.

## Materials and Methods

### Ethics Statement

This study was performed in accordance with the ethical guidelines established by The Arctic University of Norway (UiT). The project has been evaluated by the Regional Committee for Medical and Health Research Ethics, Rec North, Norway (document no. 2016/1247/REK nord), concluding that ethical approval was not required. There were no ethical issues to consider due to use of anonymous clinical samples and development of methodological procedures.

### Sample Collection

A sample collection of positive BCBs was obtained from a previous study, where we evaluated the use of Bact/Alert^®^ BCBs (bioMérieux, Marcy l’Etoile, France) for culturing PJT (Sanabria et al., 2019). In brief, three different types of BCB samples were obtained during that study: BCBs inoculated with PJT clinical samples from patients with suspicion of PJI, BCBs inoculated with tissue spiked with bacterial species reported as common microbiological causes of PJI and a negative control which was prepared by inoculating sterilized tissue (irradiated to 25 Gy) from a crushed native femoral head. For further details on the BCB sample preparation method, see Sanabria et al. (2019).

Bact/Alert^®^ BCBs were enriched with 4 mL of horse blood, which has previously been shown to produce high positivity rates and shortening of time to detection (Nylén et al., 2013) and the PJT was analyzed in parallel with the conventional diagnostic method. Bacterial identification was performed using matrix-assisted laser desorption ionization-time of flight mass spectrometry (MALDI-TOF^®^ MS Bruker Daltonics – microflex^TM^).

In this study, 25 positive aerobic BCBs (BacT/Alert FA Plus) inoculated with PJT were used initially to test two different DNA sample preparation methods. Subsequently, DNA from nine BCBs were selected for SMg. Selection criteria included: samples with presence of a single species of microorganism (monomicrobial) reported as common microbiological cause of PJI and high quality/quantity of total DNA concentration of at least 1 ng/μL bacterial DNA. Samples sequenced included: samples with *Staphylococcus aureus* (*n* = 6), *Staphylococcus epiderimidis* (*n* = 2), *Enterococcus faecalis* (*n* = 1), negative control (NC, *n* = 1), and spiked samples (positive controls, PC1-3) (*n* = 3). The negative control contained DNA extracted from a BCB enriched with horse blood and inoculated with sterilized tissue (irradiated to 25 Gy) from a donor with no suspicion of infection. The three spiked samples were BCBs inoculated with tissue spiked with a suspension of approximately 90–150 CFU/mL of *Escherichia coli* ATCC 25922, *S. aureus* ATCC 25923, or *S. aureus* ATCC 25923, and *Cutibacterium acnes* (clinical sample), respectively.

In addition, DNA from one of the nine clinical samples was sequenced three times (sample 1: S1a, S1b, S1c) in order to evaluate the impact of the sample preparation method on the metagenomic results, and to determine a suitable number of samples that could be multiplexed in one lane on the flow cell during the sequencing process.

**FIGURE 1:**
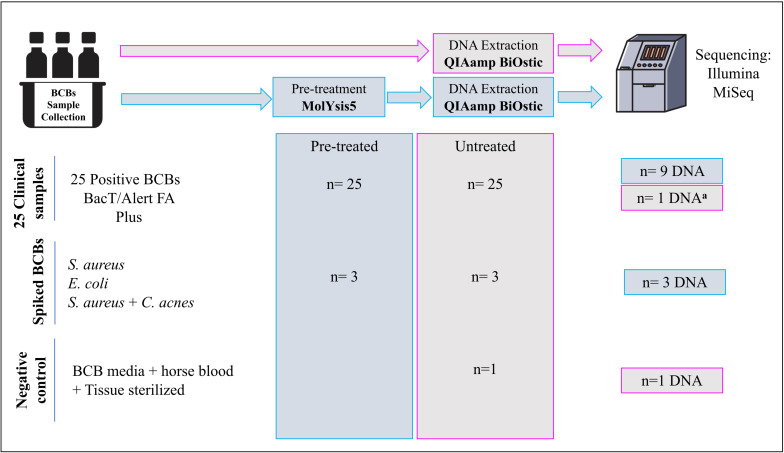
Overview of the number of samples included in each step of the process. ^*a*^DNA from a BCB sample untreated with molYsis5. Treated: BCB samples pre-treated with MolYsis5 before DNA extraction. Untreated: BCB samples with no pretreatment with MolYsis5 before DNA extraction.

Figure 1 gives an overview of the number of samples and the sample types included in each step of the process. An overview of all the samples sequenced through SMg, including the controls and their features are listed in Table 1.

**TABLE 1:** Description of samples sequenced through SMg.

Sample		Sample type inoculated in BCB	DNA preparation method	Microorganism identified Laboratory^a^ (MALDI-TOF)	
No	Code			BCB	Conventional
1	S1a	PJT (clinical sample)	MolYsis5 + BiOstic	*S. aureus*	*S. aureus*
	S1b	PJT (clinical sample)	MolYsis5 + BiOstic	*S. aureus*	*S. aureus*
	S1c	PJT (clinical sample)	BiOstic	*S. aureus*	*S. aureus*
2	S2	PJT (clinical sample)	MolYsis5 + BiOstic	*S. aureus*	*S. aureus*
3	S3	PJT (clinical sample)	MolYsis5 + BiOstic	*S. aureus*	*S. aureus*
4	S4	PJT (clinical sample)	MolYsis5 + BiOstic	*S. aureus*	*S. aureus*
5	S5	PJT (clinical sample)	MolYsis5 + BiOstic	*S. aureus*	*S. aureus*
6	S6	PJT (clinical sample)	MolYsis5 + BiOstic	*E. faecalis*	*E. faecalis*
7	S7	PJT (clinical sample)	MolYsis5 + BiOstic	*S. aureus*	No growth
8	S8	PJT (clinical sample)	MolYsis5 + BiOstic	*S. epidermidis*	No growth
9	S9	PJT (clinical sample)	MolYsis5 + BiOstic	*S. epidermidis*	No growth
10	PC1	Tissue spiked with *S. aureus*	MolYsis5 + BiOstic	*S. aureus*	NA
11	PC2	Tissue spiked with *E. coli*	MolYsis5 + BiOstic	*E. coli*	NA
12	PC3	Tissue spiked with *S. aureus* and *C. acnes*	MolYsis5 + BiOstic	*S. aureus*	NA
13	NC	Tissue sterile	BiOstic	NA	NA

**^*a*^Results from our previous study (Sanabria et al., 2019) including BCB method and conventional method. S1–S9, clinical samples; PC1-3, spiked samples (positive controls); NC, negative control; NA, not applicable.**

### DNA Preparation

Total gDNA was extracted from the blood culture bottles using the QIAamp BiOstic Bacteremia DNA Kit (Qiagen, Hilden, Germany). Samples were pre-treated using MolYsis^TM^ Basic5 kit (Molzym, Bremen, Germany) to deplete human DNA from the samples before DNA extraction. In order to find the most suitable procedure for extracting DNA from BCBs, DNA was extracted with or without pretreatment with the MolYsis5 kit. Sample pre-treatment and DNA extraction methods were according to the manufacturer’s instructions and the procedures were evaluated on the basis of DNA quantity and quality. Total DNA concentration was measured using a Qubit dsDNA HS Assay Kit (Life Technologies, Carlsbad, CA, United States) and DNA quality (OD_260_/OD_280_ and OD_260_/OD_230_) determined by Nanodrop.

### qPCR

Each DNA sample was fold diluted 1:100 and 1:1000 after determining these as suitable dilution factors to avoid inhibition during amplification. Bacterial DNA concentration was calculated by qPCR targeting the 16S rDNA gene. The standard curve consisted of a 10-fold dilution series of a mix of gDNA from bacterial species common to PJI: *S. aureus* ATCC25923, *E. coli* ATCC25922 and *S. epidermidis* ATCC 12228. The primers and the probe used were as follows: forward primer 5′-CGA AAG CGT GGG GAG CAA A-3′; reverse primer 5′-GTT CGT ACT CCC CAG GCG G-3′; probe 5′-(FAM)-ATT AGA TAC CCT GGT AGT CCA-(MGB)-3′ (Bogaert et al., 2011). The 20-μL PCR master mix consisted of 2X TaqMan Universal master mix II with UNG, 10 μM of each primer (0.8 μL), 5 μM MGB probe (0.8 μL), 2.6 μL DNA free water, and 5 μL of template DNA (Cremers et al., 2014).

Before adding the DNA template, 1M DTT and dsDNase (0.5 μL) from PCR decontamination kit (ArcticZymes, Tromsø, Norway) were added. Samples were then decontaminated by incubation at 37°C for 20 min followed by 60°C for 20 min to inactivate the dsDNase. Amplification was preformed using a 7300 Real-time PCR system (Applied Biosystems, Foster City, CA 94404 USA) under the following conditions: 50°C for 2 min, 95°C for 10 min, 50 cycles of 95°C for 15 s and 60°C for 1 min. The gDNA samples were stored at −20°C until further use.

### Metagenomic Sequencing

Sequencing libraries were prepared using the ThruPLEX^®^ DNA-seq Kit (*Rubicon* Genomics, United States) following the manufacturer’s instructions. Approximately 100 ng of DNA was used as input for library preparation from the clinical and spiked samples, while for the negative control, a little more than 1 ng was used. The sequencing process was performed at the Norwegian Sequencing Centre, Oslo, using a MiSeq sequencer (Illumina Inc., San Diego, CA, United States) with v2 chemistry and 500 cycles for 250 bp paired-end sequencing. Samples were multiplexed with 3 or 4 samples per lane. To estimate how many samples could be multiplexed in one MiSeq lane, one of the samples was run on a single lane in a pilot assay.

### Bioinformatic Data Analysis

**FIGURE 2:**
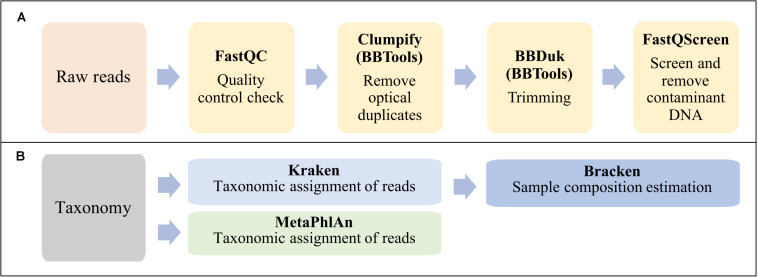
Workflow summarizing the bioinformatic analysis in this study. **(A)** Reads preprocessing. **(B)** Taxonomic classification and sample composition estimation.

The bioinformatic analysis can be summarized in two main steps: reads preprocessing (Figure 2A) and taxonomic analyses (Figure 2B).

Obtained raw reads were checked for quality using FastQC software v0.11.8^1^. Optical duplicates in fastq files were removed using the program Clumpify v38.36 from BBTools suite^2^ with default parameters. Adapter sequences were trimmed off and the poor-quality reads were removed using BBDuk of BBTools suite. The minimal length and Phred score were set to 50 and 20 nucleotides, respectively. Contaminant DNA was identified and removed by mapping all reads against the reference genomes of human GRCh38.p13 (GCF_000001405.39), horse (GCF_002863925.1) and the PhiX phage (*Escherichia* virus phiX174, GCF_000819615.1) using Bowtie2 aligner in FastQ Screen v0.13.0 (Wingett and Andrews, 2018).

Preprocessed PE reads were classified using two established tools for taxonomic profiling of metagenomic samples: Kraken (Salzberg and Wood, 2014), which is based on exact alignments of genomic k-mers, and MetaPhlAn2 which is based on clade-specific marker genes (Segata et al., 2012). However, in this work we have chosen to focus more on the Kraken analyses.

Taxonomic classification with MetaPhlAn2 v2.7.7 was executed with default parameters and using the database provided by the tool, as of July 2019. Taxonomic classification with Kraken v1.1.1 was performed using default parameters against the 8 GB dustmasked miniKraken database constructed from bacterial, archaeal, and viral genomes in Refseq, as of Oct. 18, 2017. Re-estimation of abundance was done using Bracken (Lu et al., 2017). The same pipeline for taxonomical assignment and species abundance estimation was applied to all the samples after downsizing the number of reads by randomly sampling (using SeqKit v0.11.0.) a proportion of reads from the full dataset. Subsamples from 10 to 100% of the reads were extracted and analyzed. In addition, the effect of setting a threshold for species detection level when using Kraken classifier, was evaluated by setting different threshold values (0, 10, 100, 500, 1000, 1500, 2500, 3000, 3500, and 4000 reads) on the spiked and clinical samples.

Finally, as a control to test false taxonomic assignments by Kraken, sequence reads belonging to the spiked samples and the negative control were aligned against reference genomes belonging to the top species hits from Kraken classification, using Burrows–Wheeler aligner-maximum exact matches (BWE-MEM) v0.7.17 (Li and Durbin, 2009).

### Statistical Analysis

Descriptive statistics for categorical variables were based on percentages and frequencies, while continuous variables were based on means, standard deviations (SDs), medians and interquartile ranges (IQRs). In addition, Wilcoxon rank sum test was used to evaluate if the differences between the DNA sample preparation methods were statistically significant. The differences were considered statistically significant with *P*-values lower than 0.05. Data were analyzed utilizing GraphPad Prism software, version 8.3.0 (GraphPad Software Inc., CA, United States).

Classification results from the metagenomics experiments were explored using the Pavian R package version 0.8.4 (Breitwieser and Salzberg, 2019) by using their data tables, heatmaps, and Sankey flow diagrams.

## Results

### Establishment of Method for Bacterial DNA Isolation From BCBs Inoculated With PJT

Since there is no standard procedure for the isolation of DNA from BCBs inoculated with PJT, we initially examined the performance of QIAamp BiOstic Bacteremia DNA Kit pre-treated and untreated with the MolYsis basic5 kit.

DNA from 25 positive BCBs (BacT/Alert^®^ FA Plus, aerobic bottles) were isolated using the BiOstic kit with or without pretreatment with MolYsis5. Comparison between the two methods was based on the total DNA yield and the DNA quality (Supplementary Table S1). DNA concentration measurements showed that samples treated with MolYsis5 yielded a higher mean DNA concentration (DNA 84.23 ng/μL, *p* = 0.0069), than untreated samples (65.28 ng/μL) (Supplementary Tables S2, S3). Both procedures yielded relatively pure DNA with median absorbance ratios of 1.81 (IQR = 1.67–1.90) and 1.88 (IQR = 1.8–2.02), respectively (Supplementary Figures S1A,B).

In order to evaluate the level of bacterial DNA within total DNA, qPCR was performed. Amplification on undiluted DNA extracts failed to amplify in all the cases, while amplification of DNA diluted 1:100 and 1:1000, revealed that the average bacterial DNA concentration was 21.35 ng/μL in untreated samples and 28.53 ng/μL in the MolYsis treated samples (Supplementary Figures S1C,D). All DNA samples contained at least 1 pg bacterial DNA/μL meeting the requirements needed to be considered eligible for SMg. DNA from negative controls contained 0.79% (3.6 pg/μL) and 0.04% (0.2 pg/μL) bacterial DNA without and with MolYsis treatment, respectively. The percentage of bacterial DNA was significantly different between the pre-treated and the untreated samples (*p* = 0.0207) (Supplementary Table S4). In conclusion, these studies reveal that high quality microbial gDNA was obtained from PJT on BCB samples pre-treated and untreated with MolYsis5.

### SMg Pilot Study – DNA Sample Preparation Method for Further Assays

**FIGURE 3:**
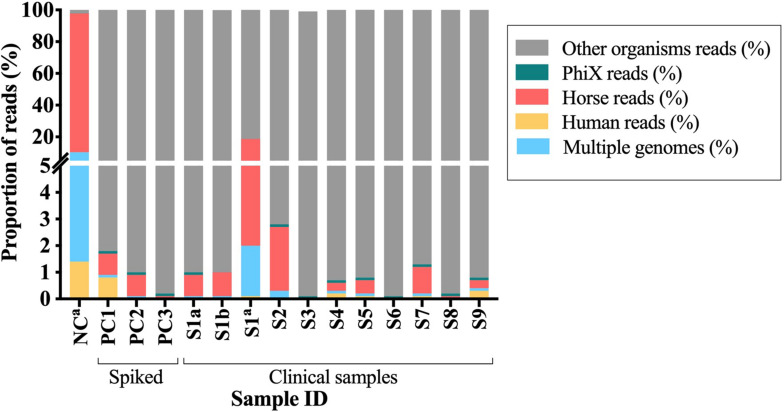
Proportion of the contaminant reads that mapped to PhiX, horse and human reference genomes or to multiple genomes. S1–S9, clinical samples; PC1-3, spiked samples (positive controls); NC, negative control. ^*a*^Samples untreated with Molysis5.

Two DNA samples (from the same BCB inoculated with a PJT clinical sample) extracted using each of the two DNA sample preparation methods [pre-treated (S1b) and untreated (S1c)], were sequenced using illumina MiSeq sequencing technology. In total, 14,785,194 and 14,078,494 raw reads were obtained from the DNA pre-treated and untreated with MolYsis5, respectively. The two samples were analyzed for the presence of contaminant DNA from human and horse sources in order to determine the proportion of contaminant reads. After preprocessing raw reads, we mapped the remaining reads against a set of human and horse reference genomes. We detected a 16-fold increase in the number of reads mapping to horse and a 19-fold increase in the number of reads mapping to human in the sample untreated with MolYsis5 (Table 2 and Figure 3).

**TABLE 2:** Reads affiliated to human and horse genomes when sample was treated (1b) and untreated with MolYsis5 (1c).

Sample code	DNA preparation method	Reads analyzed	Human reads (%)	Horse reads (%)
Treated (1b)	MolYsis5 + BiOstic	13,417,582	14,521 (0.01)	135,751 (0.9)
Untreated (1c)	BiOstic	12,698,920	249,520 (0.1)	2,365,316 (16.7)

One of the key issues in clinical metagenomics is to remove the host DNA without a substantial loss of bacterial DNA. Samples treated with MolYsis5 before DNA extraction with QIAamp BiOstic seemed to be a suitable DNA preparation method to continue with SMg from BCB.

### Shotgun-Metagenomics

Shotgun-metagenomics sequencing of DNA from clinical samples resulted in a mean number of 10,277,311 reads per sample (range 7,236,776–16,172,074). Sequencing of DNA from spiked samples produced a mean number of 9,831,669 (range 6,857,300–11,884,076) reads, and 11,192,852 total reads were obtained from sequencing the negative control (DNA without MolYsis5 treatment) (Supplementary Table S5). An additional negative control pre-treated with MolYsis5 was sent for sequencing, but no good library preparation could be obtained due to low DNA yield (results not shown). Thus, only the untreated negative control was used in the analyses.

### Extracting Sufficient Bacterial DNA While Removing DNA From Other Sources

In this study, raw reads were screened for the presence of reads from the host, from the horse blood (added to the bottles to enrich the BCB media), and from PhiX phage used as control during the sequencing process.

Overall, DNA from clinical samples presented a mean proportion of reads classified as human (0.07%), horse (0.61%), and PhiX (0.08%) lower than one percent. Similar results were observed when analyzing DNA from the spiked samples, where the mean proportion of reads mapping to the reference genomes of human were 0.26%, horse 0.56%, and to the PhiX phage 0.10%. For both cases, more than 99% of the reads did not map to any of the reference genomes used during the alignment (Figure 3). A different pattern was observed when analyzing the negative control, the mean proportion of reads mapped to human (1.4%) and horse (87.6%), while for the PhiX phage, no difference was observed (0.01%). Two percent of the reads did not map to any of the reference genomes (horse, human and PhiX) and were then used for taxonomical analyses (Figure 3 and Supplementary Table S5). Overall, samples untreated with MolYsis5 (NC and S1C) presented more reads mapping to the horse and human genomes. In addition, the proportion of reads mapping to the horse genome is higher than the human genome in all the samples (Figure 3).

After preprocessing the raw data, we obtained a mean number of 9,510,050 (92.86%) reads from the clinical samples, and 7,936,436 (86.80%) from the spiked samples, while from the negative control only 187,094 reads (1.67%) remained for taxonomical analyses (Supplementary Table S5).

Kraken was used to assign a taxonomic label to each read for estimating the relative proportion of species in the samples and to determine the relative amount of sequences that came from the known bacteria identified in the sample. Taxonomic classification by Kraken when analyzing data from clinical samples identified a median of 96.08% (IQR, 93.85–97.07%; range 85.7–98.6%) bacterial reads. Spiked samples presented a median of 98.7% (IQR, 96.90–98.70) bacterial reads, while for the negative control, the proportion of bacterial reads was 30% (28,058 reads) (Supplementary Table S6). In addition, when comparing the pre-treated (sample S1b) and the untreated sample (sample S1c) more reads were classified as bacterial reads (95.74%) for the pre-treated sample than for the untreated sample (86.89%). Overall, two percent more bacterial reads were obtained from the spiked samples than in the clinical samples. Additionally, treatment of the sample with MolYsis5 prior to DNA extraction resulted in a higher proportion of bacterial reads when compared with an untreated sample.

When estimating the number of reads classified at the genus and species level (inferring the abundance of the number of individuals from each taxon by correction for genome length into abundance estimates by Bracken), it was on average higher than 97.7% for both taxonomic levels (genus and species) and for both sample types (DNA from spiked and clinical specimens). In the negative control, 30.1% of the reads were classified to the genus and species level while the rest remained unclassified (Supplementary Table S7).

### Identification of Bacterial Species Reported as Common Cause of PJI by SMg

Nine of the PJT clinical samples in this study had previously been analyzed using a conventional and a BCB method (Sanabria et al., 2019). Bacterial identification for both methods was done using MALDI-TOF. According to the results obtained from the BCB method, *S. aureus* was identified in sample 1 (S1b), 2 (S2), 3 (S3), 4 (S4), and 7 (S7). *Enterococcus faecalis* was identified in sample 6 (S6), while in sample 8 (S8) and 9 (S9) *Staphylococcus epidermidis* was found (Table 3).

**TABLE 3:** Microorganisms identified in the samples using laboratory and SMg methods.

Sample		Microorganism identified			
		Laboratory^*a*^ (MALDI-TOF)		SMg	
No	Code	BCB	Conventional	Kraken/Bracken	MetaPhlAn2
1	S1b	*S. aureus*	*S. aureus*	*S. aureus* (99.6%)	*S. aureus* (100%)
2	S2	*S. aureus*	*S. aureus*	*S. aureus* (99.5%)	*S. aureus* (100%)
3	S3	*S. aureus*	*S. aureus*	*S. aureus* (99.9%)	*S. aureus* (100%)
4	S4	*S. aureus*	*S. aureus*	*S. aureus* (89.8%)	*S. aureus* (100%)
5	S5	*S. aureus*	*S. aureus*	*S. aureus* (99.8%)	*S. aureus* (100%)
6	S6	*E. faecalis*	*E. faecalis*	*E. faecalis* (99.9%)	*E. faecalis* (99.9%)
7	S7	*S. aureus*	No growth	*S. aureus* (99.9%)	*S. aureus* (100%)
8	S8	*S. epidermidis*	No growth	*S. epidermidis* (95.8%)	*S. epidermidis* (99.9%)
9	S9	*S. epidermidis*	No growth	*S. epidermidis* (97.7%)	*S. epidermidis* (99.9%)
10	PC1	*S. aureus*	NA	*S. aureus* (99.9%)	*S. aureus* (100%)
11	PC2	*E. coli*	NA	*E. coli* (99.7%)	*E. coli* (90.7%)
12	PC3	*S. aureus*	NA	*S. aureus* (97.3%)	*S. aureus* (100%)

**^*a*^Results from our previous study (Sanabria et al., 2019), BCB method and conventional method. PJT, Periprosthetic joint tissue; BCB, Blood culture bottle; SMg, shotgun-metagenomics; S1–S9, clinical samples; PC1-3, spiked samples (positive controls); NC, negative control; NA, not applicable.**

All bacterial species identified from the BCB culture method were detected by Kraken and MetaPhlAn, and they were found to be the most abundant species present in the sample (Supplementary Figure S2). The mean rate of the most abundant bacteria in the clinical samples were 97.9 and 99.2% for the spiked samples when using Kraken/Bracken. Similar results were found using MetaPhlAn2, with 99.2 and 96.9% mean rates for clinical and spiked samples, respectively.

**FIGURE 4:**
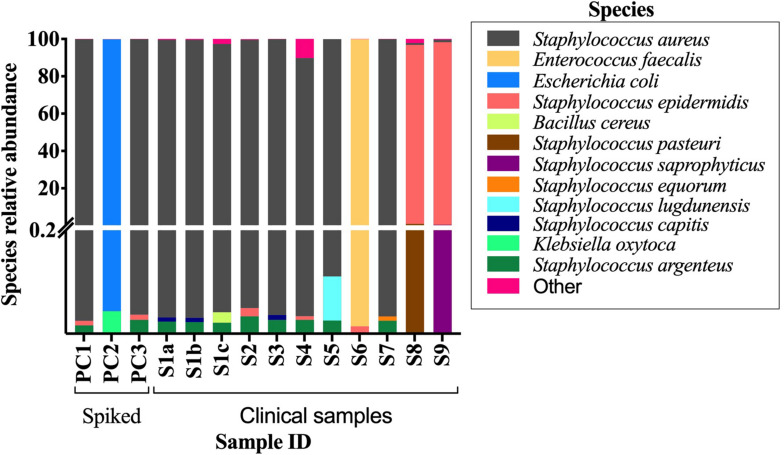
Stacked bar plot displaying relative abundance of bacteria at the species level as estimated by Kraken/Bracken in positive BCBs with PJT. Y-axis is split in two parts, and each part with their own linear scale. From 0 to 0.2 the scale highlights the small values of species relative abundance. S1-S9, clinical samples; PC1-3, spiked samples (positive controls).

The conventional culture results were all negative in samples S7–S9, while the BCB method and the SMg were consistent concerning bacterial species found (Table 3). Bacteria detected by the conventional methods and by SMg (Kraken/Bracken and MetaPhlan2) are listed in Table 3. The taxonomic classification results for each sample type excluding the negative control as estimated by Kraken/Braken are presented in Figure 4. Similarly, a heat map representing the relative abundance at the species level as estimated by MetaPhlAn2 can be found in Supplementary Figure S3.

Additionally, we tested the influence of downsizing the number of reads (by subsampling reads) in the estimation of abundance of species present in both the clinical and spiked samples. We observed that the number of species detected is higher at increasing sequencing depths and that the taxonomical distribution did not change. The potential pathogen species found in culture could still be detected even if a smaller proportion of reads were subsampled (10%). A rarefaction curve was used as a qualitative method to estimate the species abundance as a function of sequencing depth (Supplementary Figure S4). Rarefaction curve reaches plateau just for a few samples suggesting that saturation in sequencing was not achieved and deeper sequencing is required to detect all the species present. However, estimating the diversity is not the end point in PJI diagnoses.

Reads belonging to spiked samples were mapped against the reference genomes of the respective strains used for spiking. For *S. aureus* and *E. coli*, more than 98% of the genome was covered with at least 4× depth. We obtained a genome coverage depth of 775X for the sample spiked with *S. aureus* (PC1) and 209X for the sample spiked with *E. coli* (PC2). In the sample spiked with *S. aureus* and *C. acnes*, no reads were observed when mapping against *C. acnes* reference genome; instead 99.98% of the reads mapped to the *S. aureus* reference genome, covering 99.5% of its genome (Supplementary Table S11).

### Other Bacteria and Possible Contaminants Detected by SMg

In addition to the metagenomic reads belonging to the same bacteria found by the conventional methods, reads belonging to other bacterial species were also found by Kraken. Their respective abundance was estimated by Bracken in the clinical samples (mean: 1.98%; range 0.07–10.21%), and in the spiked samples (mean: 0.12%; range 0.06–0.23%). For each of the sample types, we provide the respective Sankey flow diagram with the classification results from Kraken (Supplementary Figure S5).

**FIGURE 5:**
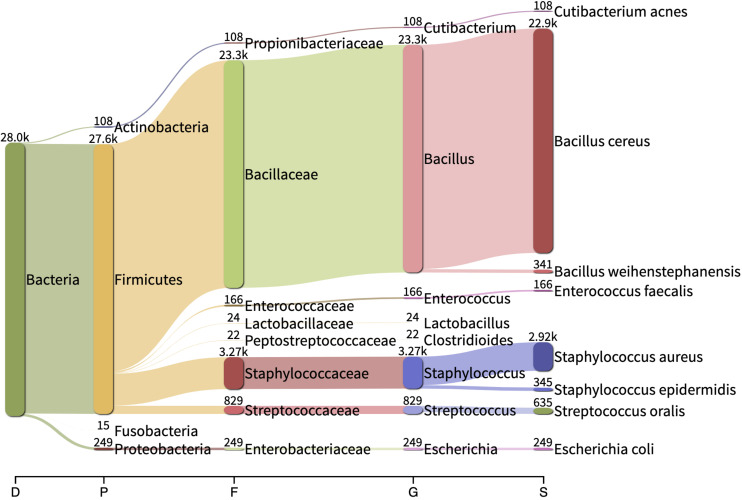
Sankey visualization of the absolute read counts as estimated by Kraken/Bracken from the negative control sample. D, Domain; P, Phylum; F, family; G, genus; D, domain.

Reads belonging to *Staphylococcus argenteus* were found in samples, S1(a, b, c), S3 S4, S5, and S7 always at the same relative abundance (0.02%) and in samples S8 and S9 at a lower abundance (0.000422 and 6.043e-05, respectively). This bacterial species was not found in the negative control (Figure 5). The most abundant bacterial species found by Kraken/Bracken in the negative control were *Bacillus cereus* (81.5%), *S. aureus* (10.3%), *Bacillus weihenstephanensis* (1.2%), and *E. coli* (0.88%) (Figure 5). MetaPhlAn did not identify *B. cereus* and *B. weihenstephanensis*, but instead identified the yeast *Saccharomyces cerevisiae* as the most abundant microorganism found in the negative control (72.5%). *S. aureus* was also found by MetaPhlAn2 at a relative abundance of 14.7%. *E. coli* was not detected but instead we found *Escherichia* unclassified (4.1%) which means “an unknown species in the genus *Escherichia.*”

When analyzing the clinical and spiked samples, we found *B. cereus* in all the samples in a very low abundance (mean, 0.0016%). It was 12-fold more abundant (0.02%) in the sample untreated with MolYsis5 (sample S1c), supporting that it is a contaminant in this study. *S. aureus* was found by Kraken/Bracken in the negative control (10.3%), as well as in samples S6, S8, S9 and in the sample spiked with *E. coli* with a very low occurrence (mean, 0.58%; range, 0.03–1.2%).

## Discussion

Here we demonstrate proof of concept that it is possible to perform SMg directly on PJT from BCBs with the potential of pathogen identification in PJI diagnosis. Culturing of PJT on BCB was the starting point for our study, and we validated our findings by comparison with conventional culturing methods, where we already knew the outcome, i.e., complete microbiological data from culturing were available for all samples.

To the best of our knowledge, this is the first study applying SMg to Bact/Alert^®^ BCBs (bioMérieux) inoculated with PJT. Sample type greatly influences the composition of sequencing reads, and due to the complexity of both the BCBs and the PJT specimens, sample preparation and bacterial enrichment methods need to be specifically adapted.

It is well-known that the success of metagenomics is highly dependent on the quality and quantity of DNA extracted from a given specimen (Schlaberg et al., 2017). Therefore, sample preparation methods need to be developed and improved to effectively reduce the amount of host nucleic acids, lyse host cells to release intracellular organisms and lyse different types of microbial cells to release nucleic acids, without affecting the quantity of targeted nucleic acid in the sample (Forbes et al., 2018; Vijayvargiya et al., 2019). Here we demonstrated that the use of MolYsis5 kit together with BiOstic kit is an effective sample preparation method for performing SMg directly from BCBs inoculated with PJT. Our DNA preparation method resulted in high quality microbial DNA from all samples, both with and without human DNA depletion, allowing downstream SMg.

Thus, we conclude that preanalytical reduction of the human DNA load improved the output of targeted sequence reads. This is important since DNA samples overwhelmed with human DNA sequences affect the sensitivity for the detection of bacteria that occur in low abundance in clinical specimens. In addition, we screened the samples and removed reads belonging to the horse genome, since horse blood was used as enrichment supplement for the BCB, which may affect the sensitivity for bacterial detection as such. Moreover, this makes the method more expensive, creates the need of subtracting the human and horse sequences during the bioinformatic analysis, which delays the analyses, and it requires a significant computational power (Hasan et al., 2016). Overall, our method consistently generated high DNA yield quantity/quality, removed efficiently human/horse DNA (<1%) and there was no inhibition observed during the SMg library preparation, allowing further sequencing process.

In order to select samples suitable for sequencing, one of the main criteria that was considered, was the bacterial DNA concentration measured by qPCR. We observed that samples had to be diluted to get amplification signals. qPCR results could not be considered as selection criteria. In addition, the concentration of bacterial DNA from qPCR did not correlate with the bacterial rate obtained from the classification of the sequencing reads (median of 96.08%). This result was not surprising since there are several factors that can affect the qPCR amplification, among them: the presence of amplification inhibitors commonly found in BCBs formulations (e.g., the anticoagulant, sodium polyaneththolesulfonate SPS) (Mäki, 2015), unspecific primer binding to host DNA which decreases the sensitivity and specificity, and the less likely in this case, due to an overload of eukaryotic DNA (since low horse and human DNA were found in the sequencing reads). However, the DNA preparation tool MolYsis which eliminates the human background, also removes other PCR-inhibiting substances contained in BCBs, like SPS (Gebert et al., 2008).

Methods for the preparation of bacterial DNA from BCBs (BD BACTEC, Becton Dickinson Sparks, MD) using SMg have been reported earlier but for bloodstream infection diagnosis (Anson et al., 2018). However, the methods included a pre-treatment by differential centrifugation to remove human cells and DNA purification with SPRI beads prior to sequencing. In their study, the use of BiOstic kit provided a higher DNA yield than Molysis Plus (430× greater), and the mean DNA yield obtained was lower (*E. coli*, 39.3 ng/μL and *S. aureus*, 11.5 ng/μL) than the DNA yield obtained in our study (Mean DNA concentration: 84.23 ng/μL for MolYsis5 treated samples and 65.28 ng/μL for untreated samples). These differences in result may be due to the type of clinical specimen inoculated into the BCBs, the effectiveness of the pre-processing and purification step, in addition to the fact that the BCBs belong to different brands.

Our results showed that MolYsis5 kit together with BiOstic kit was an effective DNA sample preparation method for the detection of the PJI related species tested here. Many bacterial and fungal species have been reported as being detectable after the use of MolYsis (Horz et al., 2010; Thoendel et al., 2016; Schmidt et al., 2017; Krohn et al., 2018). However, we are aware about potential limitations that should be taken into consideration when using MolYsis in the clinical diagnostics. Sample pre-treatment with MolYsis involves lysis of human cells prior to degradation of bacterial cell walls, allowing digestion of human DNA by DNAse treatment (Thoendel et al., 2016). The use of MolYsis provides a solution for the removal of host DNA and enrichment of intact microorganisms (Votintseva et al., 2017). However, free floating DNA is removed. This raises a question about which bacterial DNA is removed during the MolYsis treatment. Bacteria with weak or absent cell walls will be removed by the use of Molysis prior to DNA extraction. Among them, *Mycoplasma, Ureaplasma*, or *Chlamydia*, although unusual, are among the many organisms capable of causing PJIs (Geipel, 2009; Rieber et al., 2019). In addition, bacteria previously exposed to cell wall-targeting antibiotics will also be lysed and removed by the Molysis (Horz et al., 2010). This in turn raises some concerns about the proposed usefulness of MolYsis for the diagnosis of PJI when these bacteria are causing the infection. In these cases, sample pre-treatment with MolYsis should be avoided. DNA extraction with BiOstic without MolYsis pre-treatment can be used instead since good results were also obtained when using this approach in our study (sample S1c, untreated with MolYsis).

SMg for diagnosing PJI directly from tissue specimens has been tested (Ruppé et al., 2017). However, from a huge cohort of samples collected (*n* = 179), only few (*n* = 24) could be sequenced due to insufficient amounts of bacterial DNA recovered from the samples. SMg directly from sonication fluid (from orthopedic devices), has been tested as well, and this is the specimen related to PJI most extensively studied in metagenomics approaches (Thoendel et al., 2016, 2017, 2018; Street et al., 2017; Sanderson et al., 2018). Despite all these promising efforts on metagenomics for diagnosing PJI, direct sequencing of nucleic acids obtained from biological samples results in a high background of DNA, mainly derived from the host, hindering the detection of pathogens causing PJI. Thus, all these studies support clearly that the main challenge has been recovering enough bacterial DNA. Our approach was therefore to test the use of SMg directly on BCBs inoculated with PJT to try to solve the limitations observed when using SMg directly on PJI specimens.

In our study, predominant bacterial species in PJT from BCBs determined by SMg, were 100% concordant with the results obtained from the BCB culture method. Results were consistent with respect to both the genus and species levels. We were able to detect *S. aureus, S. epidermidis, E. faecalis, and E. coli* in the samples, indicating the potential of the method for detection of species commonly related to be the cause of PJI. The predominant species (mean rate: 97.9%), reads belonging to other bacterial species were also found by the taxonomical classifier in a very low proportion (mean rate: 1.98%).

Apart from the development and/or improvement in the DNA sample preparation methods, one of the greatest challenges in the use of SMg for identification of pathogens is the type of controls (Couto et al., 2018). Positive controls should represent the range of organisms that can be encountered in the clinical specimen (Greninger and Naccache, 2019). In this study, three spiked samples were included as positive controls. Two of them spiked with one bacterial species (*S. aureus* or *E. coli*) and one with two bacterial species (*S. aureus* and *C. acnes*). High breadth and depth coverage were obtained when mapping the SMg reads to *S. aureus* or *E. coli* reference genomes, respectively. However, *C. acnes* was not found in the SMg taxonomical analyses of the spiked control sample. It could be several reasons for this, but we believe that it was due to mistakes in the experimental design. *S. aureus* and *C. acnes* were spiked into the BCB at the same time and we know from our previous study (Sanabria et al., 2019) that *C. acnes* grows slower (mean time to detection: 8.7 days) than *S. aureus* in the Bact/Alert^®^ BCBs. The positive control was incubated in an aerobic BCB until positive, i.e., in this case 10.3 h after incubation. We believe that the absence of reads belonging to *C. acnes* is due to the fact that the bacterium did not have enough time to grow. In addition, *S. aureus* might be a strong competitor. Another possible explanation could be incomplete lysis of *C. acnes* during the DNA sample preparation and the lack of sensitivity of the SMg to detect the anaerobic *C. acnes*.

For the case of the negative control, we used a BCB medium enriched with horse blood and inoculated with sterilized tissue from a donor without suspicion of infection. We consider that this negative control adequately reflects the contaminant or background microorganisms originating from tissue specimens, BCB media, horse blood, reagents, the environment and other sources as from other samples and sequencing runs (Eisenhofer et al., 2019; Huang et al., 2019). Results showed that from 93,502 reads, only 28,194 could be classified (30%).

In the negative control, we found taxa reported as common contaminants (DNA extraction blank controls and no-template controls) (Eisenhofer et al., 2019), e.g., *Bacillus*, *Staphylococcus*, *Enterococcus* and *Streptococcus*. We also found some reads classified as *B. cereus* in several of the clinical samples (in a very low abundance, mean: 0.0016%) and this bacterium was found 12-fold more abundant (0.02%) in the sample not treated with Molysis5 (sample S1c). *Bacillus* spp. is often considered a contaminant when it is isolated from BCBs and in negative controls (Doern et al., 2020). However, the significance of this in SMg on samples from BCBs is unknown.

We also found some reads assigned to *S. aureus* (10.3%) and *E. coli* (0.88%) in the negative control. Since these bacteria are among the most common causes of PJI (Tande and Patel, 2014; Izakovicova et al., 2019), these reads were evaluated by aligning the reads against the reference genomes of the strain with the highest assignment number of reads. When visualizing the mapping results, reads mapped with genetic areas belonging to coding sequences annotated as RNAs with a very low coverage depth, and they were not distributed all over the genomes. These may be reads originating from laboratory, *in silico* or kit contaminants. Contamination is one of the main concerns in PJI diagnostics and even more in metagenomic sequencing.

Reports have demonstrated that even the commercial kits for DNA extraction and library preparation are potential for contamination leading to misinterpretation of sequencing data from clinical specimens (Salter et al., 2014; Eisenhofer et al., 2019). It is therefore recommended to include and sequence negative controls when performing SMg studies.

The spectrum of organisms defined as reportable by SMg assays should be defined, and organisms determined to be background contaminants or clinically insignificant should be described (Gu et al., 2019). Defining a contaminant is not clear for blood cultures in the laboratory and present a challenge for SMg (Greninger and Naccache, 2019). Many factors should be considered when interpreting the results from SMg especially because the results are highly dependent on the database used for the analysis, which could be incomplete for rare pathogens or biased toward certain organisms, in addition to the fact that contamination with normal flora and reagents are a common occurrences that can limit specificity (Gu et al., 2019). Consequently, it is very important to be careful when analyzing the clinical significance of the results.

The most common *in silico* decontamination method in practice is the removal of sequences below a determined detection threshold (Davis et al., 2018). Usually, software used for taxonomical classification such as Kraken can predict a lot of species. Although we have limited clinical data to distinguish between true PJI and contamination, we tested the effect of several thresholds on the estimation of species abundance in the samples. We observed that the number of species detected in the samples are highly dependent on the threshold value used during the analyses (Supplementary Figure S6 and Supplementary Table S10). The results presented here prove that for species detection, thresholds may often lead to different inferences while interpreting the diagnostic results. Consequently, when using SMg, thresholds need to be validated in each specific case for an accurate interpretation of the results (Schlaberg et al., 2017). In our case, unfortunately we cannot allow to give an exact cut-off value, due to limited access to clinical data of our samples. However, others have set optimal thresholds for differentiating low-level contaminations from true PJI when using SMg on sonication fluid (Street et al., 2017; Ivy et al., 2018).

There is no standard method for interpreting metagenomic sequencing results. Contaminant DNA in SMg is a challenge for clinical interpretation of metagenomics data (Peel et al., 2016; Thoendel et al., 2016; Ruppé et al., 2017; Street et al., 2017; Thoendel et al., 2017; Ivy et al., 2018; Simner et al., 2018; Thoendel et al., 2018). We cannot exclude the possibility of contamination in our study. As in most studies, all our samples contained read identifications for microorganisms other than known or suspected pathogens. In order to determine if the bacterial species found are infection inducing pathogens, background contaminants or noise, we observed that there are several aspects that can help to differentiate them, among them: (i) The proportion of reads assigned to the species present. The possibility of obtaining quantified abundances of microorganisms is important for distinguishing causative pathogens (Greninger and Naccache, 2019). In our case, we considered that a high proportion of reads belonging to the most abundant bacteria present in the samples (97.9% mean rate) could be an indicator of the bacterium causing the infection. (ii) Genome coverage and the proportion of the genome covered, higher depth and breadth coverage expected for the pathogen species with respect to other species detected by the taxonomical classifier. For pathogen detection it is even more important because sequencing depth also affects analytic sensitivity (Schlaberg et al., 2017; Couto et al., 2018). (iii) Comparison with respect to the species and to the proportions of reads found in the negative control and in the spiked samples, and (iv) To set an appropriate threshold, for pathogen detection. Regarding this, it is important to limit the number of species identified, for minimizing false-positive results, increasing the detection rate of potential true pathogens and reducing the misclassification of other species related signals as potential pathogens (Couto et al., 2018). It is important to consider that setting up cut-off values for pathogen detection may result in decreased sensitivity. Therefore, it is better to rely on the relative abundance of bacterial species in addition to the genome coverage and the proportion of the genome covered.

It is important to predict the level at which samples should be sequenced to prevent excessive sequencing and to answer our biological question (Sims et al., 2014). The relatively high cost for metagenomic sequencing is a major limitation for application in the clinical setting (Ruppé et al., 2017). Significant reduction in the cost of metagenomic sequencing is required for moving up in the diagnostic pipeline (Greninger, 2018). Multiplexing samples offers the possibility of decreasing the costs by decreasing the number of reads per sample. The question is how many reads are needed to answer a particular question (Mulcahy-O’Grady and Workentine, 2016). In this study, we wanted to assess the potential of SMg for the detection of bacterial species known as common causes of PJI. We analyzed the effect of reduction in sequencing depth (expressed as proportion of reads) for the detection of potential PJI pathogens and, we observed that even while using the minimal proportion of reads subsampled (10%) we were able to detect *S. aureus* (Supplementary Figure S7). This result suggests that presumably less sequencing depth is needed for detection of common PJI pathogens, more samples can be multiplexed in a sequencing lane, accomplishing a lower cost per sample. However, it is important to be aware that the impact of a lower sequencing depth to provide additional information beyond pathogen detection was not considered in this study. Nevertheless, the results obtained in this study open the possibility for studying antibiotic resistance determinants and virulence genes at further stages.

Our study has the following technical limitations: **(1)** Low total number of clinical samples analyzed trough shotgun-metagenomics (*n* = 9) and just one sample per patient. **(2)** Limited clinical data about the patients making it difficult to define a sample selection criterion to distinguish between true PJI and contamination; **(3)** Negative samples by conventional and BCB culture methods were not included. **(4)** Only aerobic BCBs were included. **(5)** Absence of polymicrobial samples (all samples tested were monomicrobial by culture). **(6)** In comparison with the conventional and the BCB culture methods, the application of SMg is limited by the expensive equipment and operational costs.

The use of clinical metagenomics approaches will increase during the next years in research and in medical microbiology laboratories (Deurenberg et al., 2017). The application in clinical microbiology is still in its infancy, which encourage further research on alternative and complementary tools for PJI diagnosis. There are ongoing discussions about the obstacles associated with the adoption of metagenomics in diagnostics and their clinical utility (Greninger, 2018; Chiu and Miller, 2019; Han et al., 2019). However, we do not believe that SMg can replace conventional culturing, but it can be a potential diagnostic tool to support conventional culture in cases when PJI diagnosis is challenging, e.g., with fastidious organisms, discrepancies between conventional methods, or in culture negative cases.

In conclusion, our DNA preparation method resulted in high quality microbial DNA from all PJT samples, both with and without human DNA depletion, allowing downstream SMg. By SMg we were able to identify relevant PJI pathogens, and all bacteria identified by culture were also identified through SMg. A high enough sequencing depth was obtained indicating that it is possible to multiplex samples reducing costs considerably. We achieved a high sequencing quality, low human DNA content, high number of reads and complete genome coverage of sufficient depth that technically can be used for AMR prediction, virulence gene detection and bacterial typing.

We consider this an essential step in further studies for solving the challenges presented in PJI diagnosis, e.g., when bacteria are not detected by the laboratory methods but there is still clinical signs of the presence of infection (Peel et al., 2016). It is still possible to extract DNA from a negative BCB and analyze if pathogenic bacteria are present. In fact, the results of SMg can also be valuable even when concordant with laboratory results, not only providing a guarantee that the laboratory diagnosis is correct, but also allowing extra information, e.g., detecting coinfections and/or predicting antimicrobial susceptibility. Our results can be useful for further validation and standardization for the use of SMg on BCBs inoculated with clinical samples for routine diagnostics of pathogens. It is still a long way until SMg can be used in the clinical microbiology laboratory, but this SMg approach presents an alternative tool in PJI diagnosis, complementing the currently available tools.

## Data Availability Statement

The datasets generated for this study can be found in the European Nucleotide Archive repository (ENA) (www.ebi.ac.uk/ena), with the study accession number PRJEB36855.

## Ethics Statement

This study was performed in accordance with the ethical guidelines established by the Arctic University of Norway (UiT). The project has been evaluated by the Regional Committee for Medical and Health Research Ethics, Rec North, Norway (document no. 2016/1247/REK nord), concluding that ethical approval was not required. There were no ethical issues to consider due to use of anonymous clinical samples and development of methodological procedures. Written informed consent for participation was not required for this study in accordance with the national legislation and the institutional requirements.

## Author Contributions

AS performed the experiments and the bioinformatic analysis, took part in the study design, and wrote the first version of the manuscript. AS, MJ, JS, GS, and A-MH assisted in the scientific and technical design of the experiments. EH gave advice on the bioinformatic analyzes and revised the manuscript. AS, A-MH, and GS analyzed and interpreted the data results. All authors contributed to the article and approved the submitted version.

## Conflict of Interest

The authors declare that the research was conducted in the absence of any commercial or financial relationships that could be construed as a potential conflict of interest.

## Footnotes

1 http://www.bioinformatics.babraham.ac.uk/projects/fastqc/

2 https://jgi.doe.gov/data-and-tools/bbtools/
