# Supplementary material for: Shotgun-Metagenomics on Positive Blood Culture Bottles Inoculated With Prosthetic Joint Tissue: A Proof of Concept Study
Source: Front Microbiol. 2020 Jul 17;11:1687. doi: 10.3389/fmicb.2020.01687 (PMC7380264; doi:10.3389/fmicb.2020.01687)
Supplement: Supplementary file 1 [file Table_1.DOCX]

| **Sample No** | **DNA sample preparation methods** | | | | | | | | | |
| --- | --- | --- | --- | --- | --- | --- | --- | --- | --- | --- |
|  | **Method: BiOstic Kit** | | | | | **Method: MolYsis+BiOstic** | | | | |
|  | **Qubit**  **ng/µl** | **Nanodrop**  **ng/µl** | **Abs**  **260/280** | **Abs**  **260/230** | **Bacterial**  **DNA ng/µl** | **Qubit**  **ng/µl** | **Nanodrop**  **ng/µl** | **Abs**  **260/280** | **Abs**  **260/230** | **Bacterial**  **DNA ng/µl** |
| **1** | 27,6 | 23,5 | 1,88 | 1,85 | 0,22 | 75,4 | 81 | 1,8 | 1,97 | 4,2 |
| **2** | 5,8 | 6,5 | 2,06 | 1,04 | 1,7 | 27 | 45,8 | 1,94 | 2,22 | 7,8 |
| **3** | 94,6 | 70,3 | 1,86 | 2,17 | 0,058 | 114 | 213 | 1,7 | 1,4 | 4,26 |
| **4** | 80 | 103,5 | 1,83 | 2,11 | 4,57 | 62 | 111 | 1,68 | 1,49 | 60,15 |
| **5** | 110 | 104,4 | 1,77 | 1,85 | 19 | 98 | 103,8 | 1,77 | 1,81 | 45 |
| **6** | 110 | 101,6 | 1,8 | 1,95 | 49 | 116 | 484,6 | 2,04 | 2,29 | 50 |
| **7** | 59,2 | 42,5 | 1,86 | 2,21 | 17,3 | 99,8 | 433,6 | 2,08 | 2,3 | 16,3 |
| **8** | 94,4 | 81,9 | 1,87 | 2,32 | 51 | 114 | 109,7 | 1,82 | 1,97 | 43 |
| **9** | 83,4 | 540,1 | 2,06 | 2,27 | 66 | 94,8 | 610 | 1,98 | 2,18 | 74,1 |
| **10** | 52,2 | 272,7 | 2,09 | 2,2 | 25 | 75,4 | 259,8 | 1,96 | 2,03 | 45,2 |
| **11** | 62,6 | 494,6 | 2,07 | 2,26 | 27,1 | 74,6 | 151,3 | 1,85 | 1,99 | 30,9 |
| **12** | 55 | 218,6 | 1,99 | 2,27 | 29 | 68 | 138,4 | 1,88 | 1,96 | 20,61 |
| **13** | 55,6 | 383,8 | 2,09 | 2,27 | 3,19 | 74,4 | 134,7 | 1,83 | 2,04 | 30,3 |
| **14** | 66,8 | 472,9 | 2,1 | 2,33 | 33 | 78,4 | 162,2 | 1,85 | 2,01 | 50,8 |
| **15** | 17,1 | 62,7 | 1,91 | 1,77 | 7,2 | 16,6 | 34,8 | 1,66 | 1,23 | 13,7 |
| **16** | 65,2 | 178,4 | 1,93 | 2,38 | 25,93 | 78,4 | 295,1 | 1,81 | 1,96 | 29,8 |
| **17** | 66,8 | 191,2 | 1,95 | 2,4 | 29 | 77,2 | 298,9 | 1,79 | 1,96 | 0,54 |
| **18** | 86,8 | 82,2 | 1,7 | 1,21 | 11,7 | 100 | 149,2 | 1,78 | 1,42 | 17,2 |
| **19** | 51,6 | 100,2 | 1,36 | 0,85 | 24,2 | 49 | 143,2 | 1,68 | 1,32 | 23,73 |
| **20** | 66,8 | 115,6 | 1,79 | 1,8 | 22 | 47 | 106,9 | 1,56 | 1,07 | 55,47 |
| **21** | 60,6 | 65,1 | 1,82 | 2,03 | 13,8 | 47,8 | 65,1 | 1,36 | 0,85 | 21,5 |
| **22** | 36 | 16,8 | 1,8 | 1,59 | 1,88 | 110 | 67,4 | 1,92 | 1,92 | 5,82 |
| **23** | 91 | 65,4 | 1,85 | 2,04 | 7,39 | 245 | 193,3 | 1,81 | 1,63 | 41,3 |
| **24** | 118 | 216,7 | 1,9 | 2,23 | 63,7 | 104 | 110,5 | 1,49 | 0,98 | 6 |
| **25** | 15 | 32,4 | 1,65 | 0,95 | 0,78 | 59 | 65 | 1,67 | 1,56 | 15,6 |

**Supplementary Table S1.** DNA Extraction results obtained from the two sample preparation methods tested.
