# Supplementary material for: Shotgun-Metagenomics on Positive Blood Culture Bottles Inoculated With Prosthetic Joint Tissue: A Proof of Concept Study
Source: Front Microbiol. 2020 Jul 17;11:1687. doi: 10.3389/fmicb.2020.01687 (PMC7380264; doi:10.3389/fmicb.2020.01687)
Supplement: Supplementary file 2 [file Table_2.DOCX]

| **Parameter** | **BiOstic** | **MolYsis5 + BiOstic** |
| --- | --- | --- |
| **Number of values** | 25 | 25 |
|  |  |  |
| **Minimum** | 5,8 | 16,6 |
| **25% Percentile** | 51,9 | 60,5 |
| **Median** | 65,2 | 77,2 |
| **75% Percentile** | 88,9 | 102 |
| **Maximum** | 118 | 245 |
| **Range** | 112,2 | 228,4 |
|  |  |  |
| **10% Percentile** | 16,26 | 39 |
| **90% Percentile** | 110 | 114,8 |
|  |  |  |
| **Mean** | 65,28 | 84,23 |
| **Std. Deviation** | 29,9 | 42,88 |
| **Std. Error of Mean** | 5,979 | 8,577 |
|  |  |  |
| **Coefficient of variation** | 45.79% | 50.91% |
|  |  |  |
| **Sum** | 1632 | 2106 |

**Supplementary Table S2.** Descriptive statistics from the total DNA concentration results obtained from the two sample preparation methods tested.
