# Supplementary material for: Shotgun-Metagenomics on Positive Blood Culture Bottles Inoculated With Prosthetic Joint Tissue: A Proof of Concept Study
Source: Front Microbiol. 2020 Jul 17;11:1687. doi: 10.3389/fmicb.2020.01687 (PMC7380264; doi:10.3389/fmicb.2020.01687)
Supplement: Supplementary file 3 [file Table_3.DOCX]

**Supplementary Table S3.** Results from the statistical test (Wilcoxon rank sum test) applied to the total DNA concentration results obtained, for evaluating the statistical significance amongst the two sample preparation methods tested.

| **Table Analyzed** | DNA concentration |
| --- | --- |
|  |  |
| **Column B** | MolYsis5 + BiOstic |
| **vs.** | vs. |
| **Column A** | BiOstic |
|  |  |
| **Wilcoxon matched-pairs signed rank test** |  |
| **P value** | **0,0069** |
| **Exact or approximate P value?** | Exact |
| **P value summary** | ** |
| **Significantly different (P < 0.05)?** | Yes |
| **One- or two-tailed P value?** | Two-tailed |
| **Sum of positive, negative ranks** | 260.5 , -64.50 |
| **Sum of signed ranks (W)** | 196 |
| **Number of pairs** | 25 |
| **Number of ties (ignored)** | 0 |
|  |  |
| **Median of differences** |  |
| **Median** | 13 |
|  |  |
| **How effective was the pairing?** |  |
| **rs (Spearman)** | 0,6837 |
| **P value (one tailed)** | <0.0001 |
| **P value summary** | **** |
| **Was the pairing significantly effective?** | Yes |
