# Supplementary material for: Shotgun-Metagenomics on Positive Blood Culture Bottles Inoculated With Prosthetic Joint Tissue: A Proof of Concept Study
Source: Front Microbiol. 2020 Jul 17;11:1687. doi: 10.3389/fmicb.2020.01687 (PMC7380264; doi:10.3389/fmicb.2020.01687)
Supplement: Supplementary file 4 [file Table_4.DOCX]

**Supplementary Table S4.** Results from the statistical test (Wilcoxon rank sum test) applied to the bacterial DNA concentration results obtained by qPCR, for evaluating the statistical significance amongst the two sample preparation methods tested.

| **Table Analyzed** | qPCR_Bacterial_DNA |
| --- | --- |
|  |  |
| **Column B** | MolYsis5 +  BiOstic |
| **vs.** | vs. |
| **Column A** | BiOstic |
|  |  |
| **Wilcoxon matched-pairs signed rank test** |  |
| **P value** | **0,0207** |
| **Exact or approximate P value?** | Exact |
| **P value summary** | * |
| **Significantly different (P < 0.05)?** | Yes |
| **One- or two-tailed P value?** | Two-tailed |
| **Sum of positive, negative ranks** | 247.5 , -77.50 |
| **Sum of signed ranks (W)** | 170 |
| **Number of pairs** | 25 |
| **Number of ties (ignored)** | 0 |
|  |  |
| **Median of differences** |  |
| **Median** | 5,5 |
|  |  |
| **How effective was the pairing?** |  |
| **rs (Spearman)** | 0,4493 |
| **P value (one tailed)** | 0,0121 |
| **P value summary** | * |
| **Was the pairing significantly effective?** | Yes |
