# Supplementary material for: Shotgun-Metagenomics on Positive Blood Culture Bottles Inoculated With Prosthetic Joint Tissue: A Proof of Concept Study
Source: Front Microbiol. 2020 Jul 17;11:1687. doi: 10.3389/fmicb.2020.01687 (PMC7380264; doi:10.3389/fmicb.2020.01687)
Supplement: Supplementary file 8 [file Table_8.DOCX]

**Supplementary Table S8.** Number of Species and genera determined by Kraken when no threshold was considered. S1-S9: clinical samples, PC1-3: spiked samples (positive controls), NC: negative control.

| **Sample** | **Number of species** | **Number of genera** |
| --- | --- | --- |
| S1b | 98 | 36 |
| S2 | 113 | 51 |
| S3 | 64 | 23 |
| S4 | 105 | 33 |
| S5 | 82 | 32 |
| S6 | 75 | 37 |
| S7 | 99 | 55 |
| S8 | 71 | 34 |
| S9 | 53 | 18 |
| PC1 | 126 | 62 |
| PC2 | 121 | 53 |
| PC3 | 71 | 27 |
| NC | 107 | 78 |
