# Supplementary material for: Shotgun-Metagenomics on Positive Blood Culture Bottles Inoculated With Prosthetic Joint Tissue: A Proof of Concept Study
Source: Front Microbiol. 2020 Jul 17;11:1687. doi: 10.3389/fmicb.2020.01687 (PMC7380264; doi:10.3389/fmicb.2020.01687)
Supplement: Supplementary file 9 [file Table_9.DOCX]

**Supplementary Table S9.** Descriptive statistics from the Number of Species and genus determined by Kraken when no threshold was considered. CS: clinical samples, all: clinical samples and spiked samples (positive controls).

|  | **Number of**  **species_all** | **Number of**  **genera_all** | **Species**  **CS** | **Genera**  **CS** |
| --- | --- | --- | --- | --- |
| Number of values | 13 | 13 | 9 | 9 |
|  |  |  |  |  |
| Minimum | 53 | 18 | 53 | 18 |
| 25% Percentile | 71 | 29,5 | 67,5 | 27,5 |
| Median | 98 | 36 | 82 | 34 |
| 75% Percentile | 110 | 54 | 102 | 44 |
| Maximum | 126 | 78 | 113 | 55 |
| Range | 73 | 60 | 60 | 37 |
|  |  |  |  |  |
| Mean | 91,15 | 41,46 | 84,44 | 35,44 |
| Std. Deviation | 23,24 | 17,15 | 20,36 | 11,76 |
| Std. Error of Mean | 6,447 | 4,758 | 6,787 | 3,92 |
|  |  |  |  |  |
| Coefficient of variation | 25.50% | 41.37% | 24.11% | 33.18% |
|  |  |  |  |  |
| Sum | 1185 | 539 | 760 | 319 |
