# Supplementary material for: Shotgun-Metagenomics on Positive Blood Culture Bottles Inoculated With Prosthetic Joint Tissue: A Proof of Concept Study
Source: Front Microbiol. 2020 Jul 17;11:1687. doi: 10.3389/fmicb.2020.01687 (PMC7380264; doi:10.3389/fmicb.2020.01687)
Supplement: Supplementary file 11 [file Table_11.DOCX]

**Supplementary Table S11.** Results from mapping of spiked sample reads against reference genomes. PC1-PC3: Spiked samples (positive controls).

| **Sample** | **Reference**  **genome** | **Reference**  **Genome NCBI accession number** | **Lenght of reference genome** | **Total**  **reads** | **Mapped**  **and**  **paired**  **reads** | **Unmapped reads** | **Total number**  **of covered**  **bases^a^** | **Coverage breadth**  **(%)** | **Coverage depth** |
| --- | --- | --- | --- | --- | --- | --- | --- | --- | --- |
| **PC1** | ***S. aureus* ATCC 25923** | GCF_000756205.1 | 2 806 346 | 10 456 806 | 10 455 042 | 1 765 | 2 806 306 | 99,7 | 775X |
| **PC2** | ***E. coli***  **ATCC 25922** | GCF_000743255.1 | 5 203 440 | 5 321 658 | 5 264 048 | 56 178 | 5 203 372 | 98,6 | 209X |
| **PC3** | ***S. aureus* ATCC 25923** | GCF_000756205.1 | 5 203 440 | 8 030 844 | 8 029 324 | 1 440 | 2 806 345 | 99,5 | 583X |

**^a^** Total number of covered bases with a coverage depth bigger or equal to 4X. PC1-3: spiked samples (positive controls).
