# Supplementary figures and images for: Shotgun-Metagenomics on Positive Blood Culture Bottles Inoculated With Prosthetic Joint Tissue: A Proof of Concept Study
Source: Front Microbiol. 2020 Jul 17;11:1687. doi: 10.3389/fmicb.2020.01687 (PMC7380264; doi:10.3389/fmicb.2020.01687)

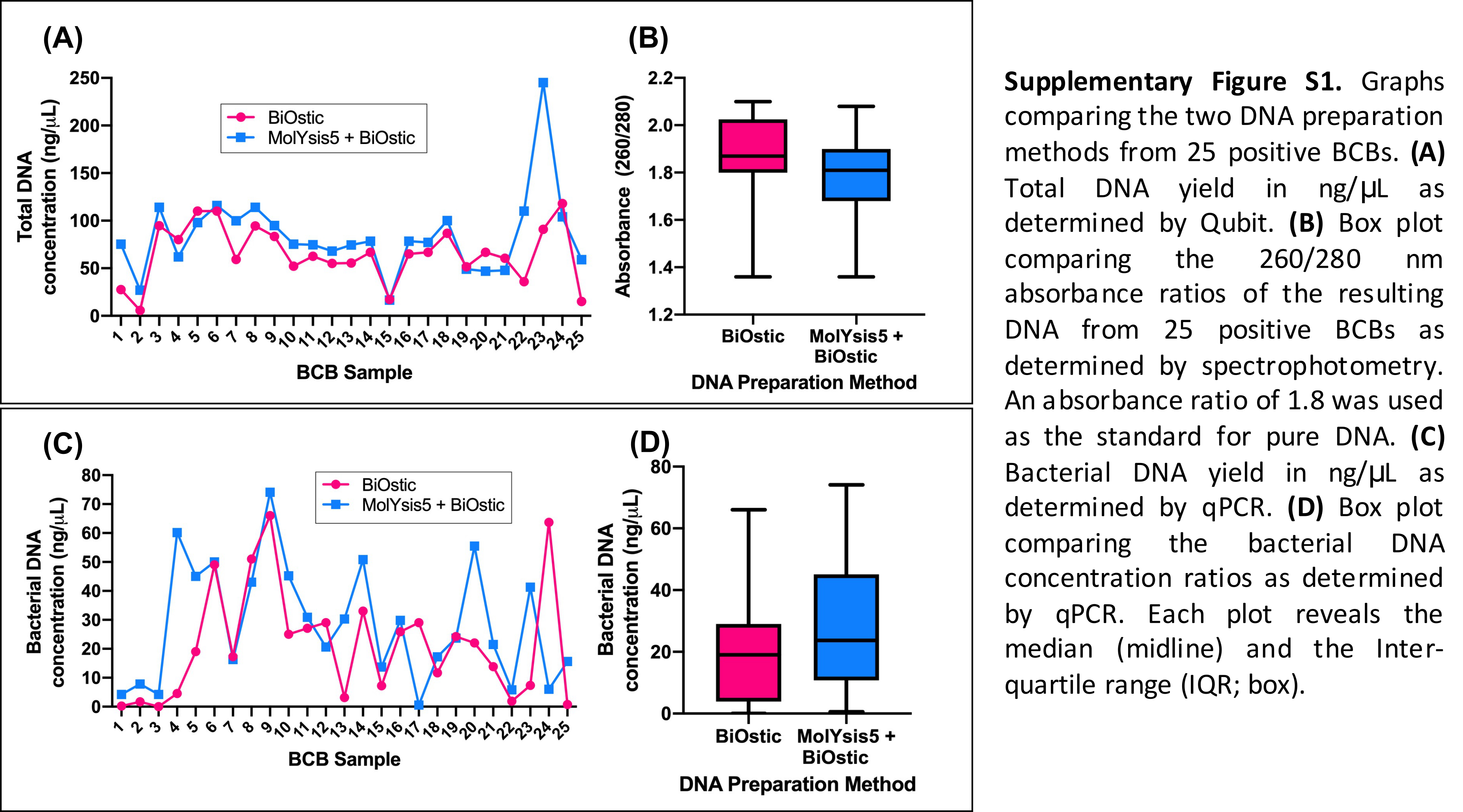

Supplement: Supplementary file 12 [file Image_1.JPEG]

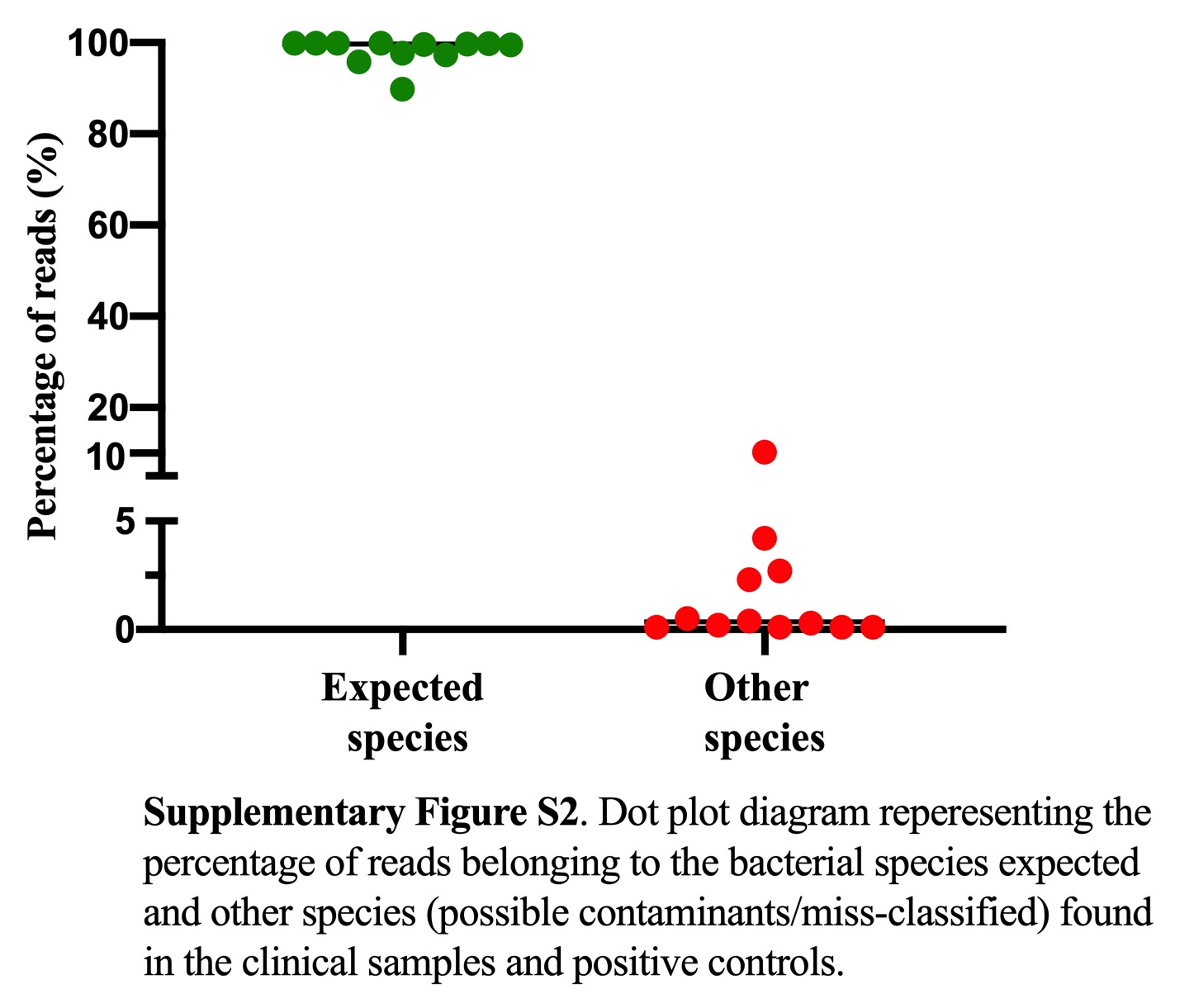

Supplement: Supplementary file 13 [file Image_2.JPEG]

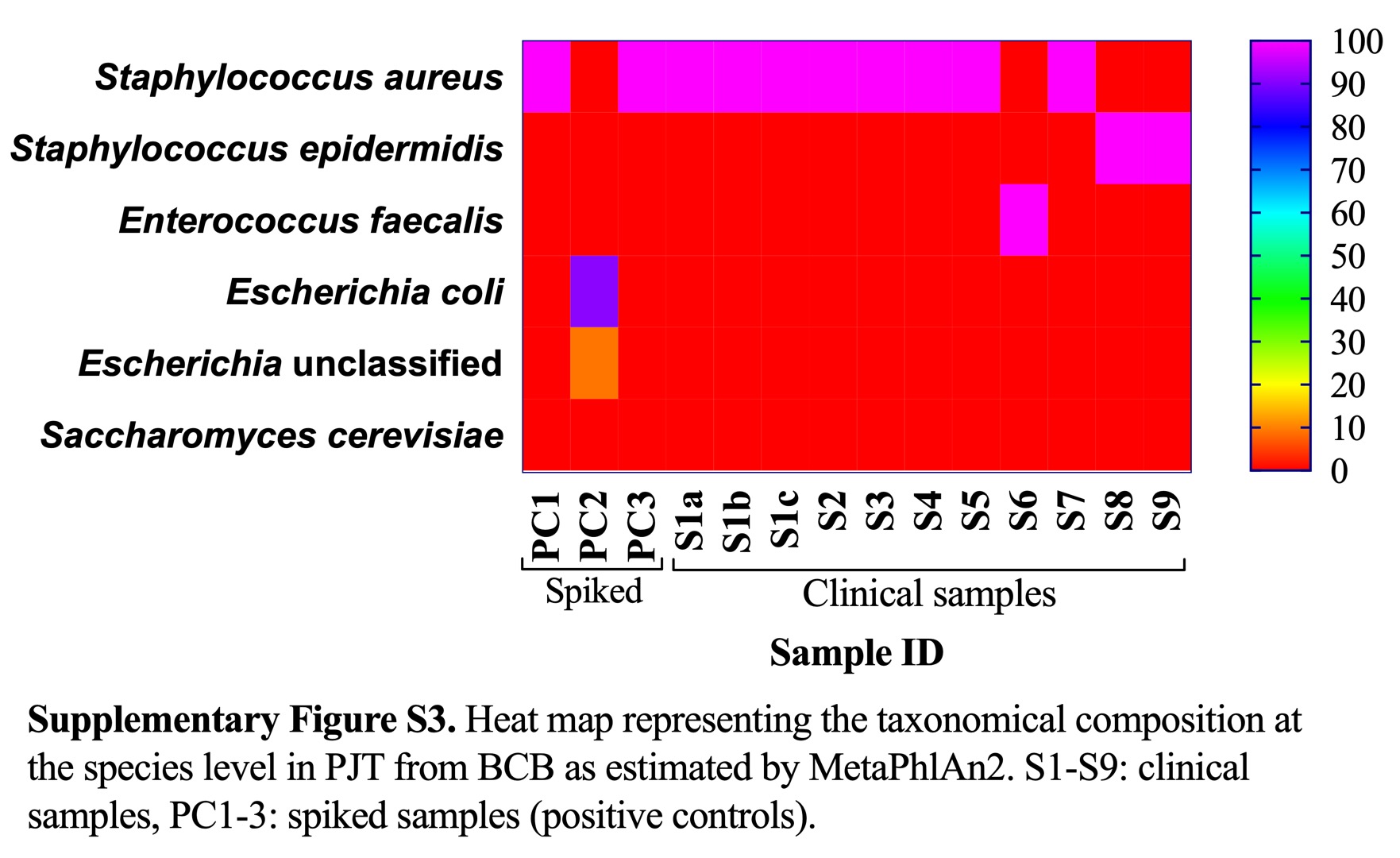

Supplement: Supplementary file 14 [file Image_3.JPEG]

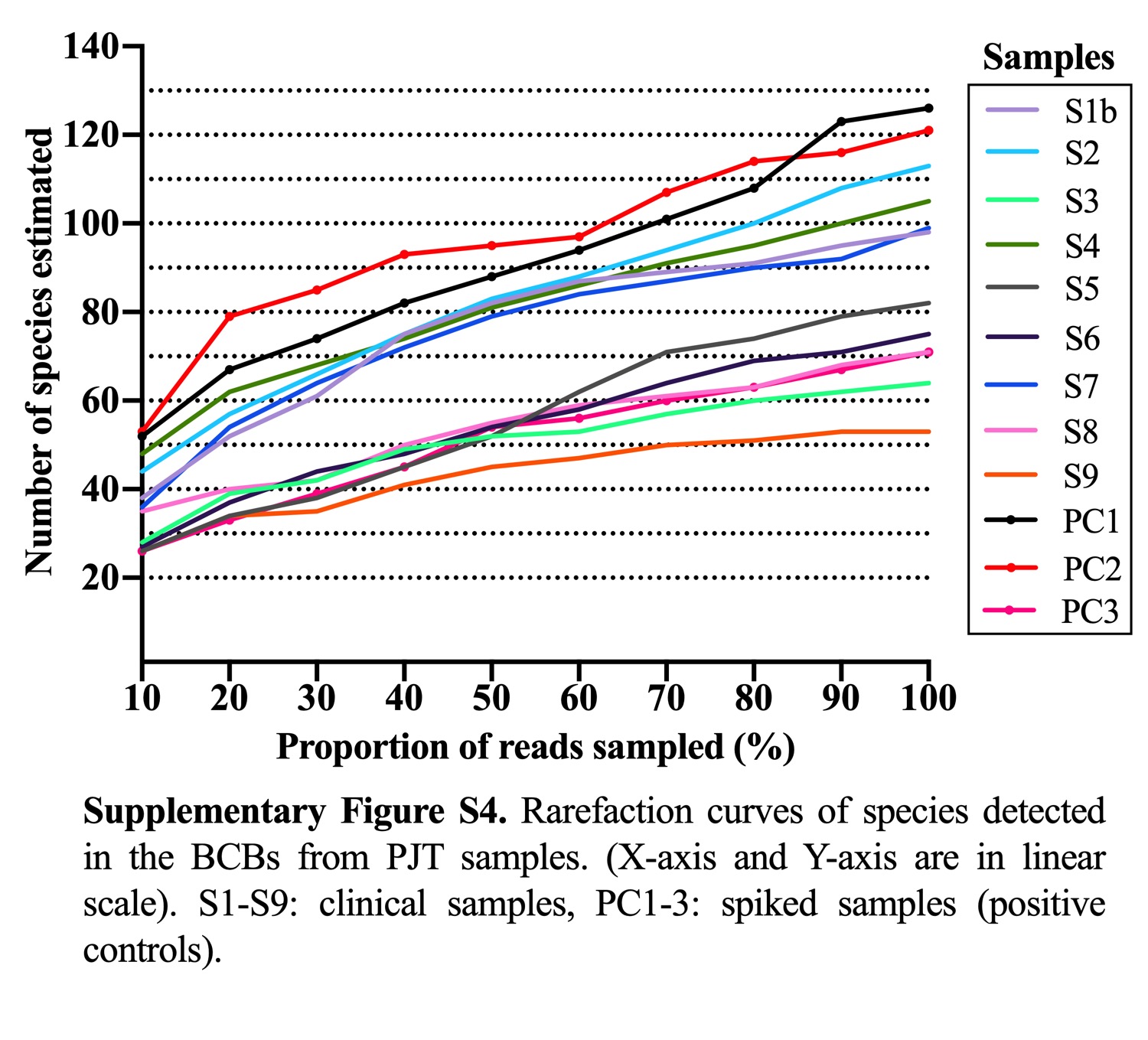

Supplement: Supplementary file 15 [file Image_4.JPEG]

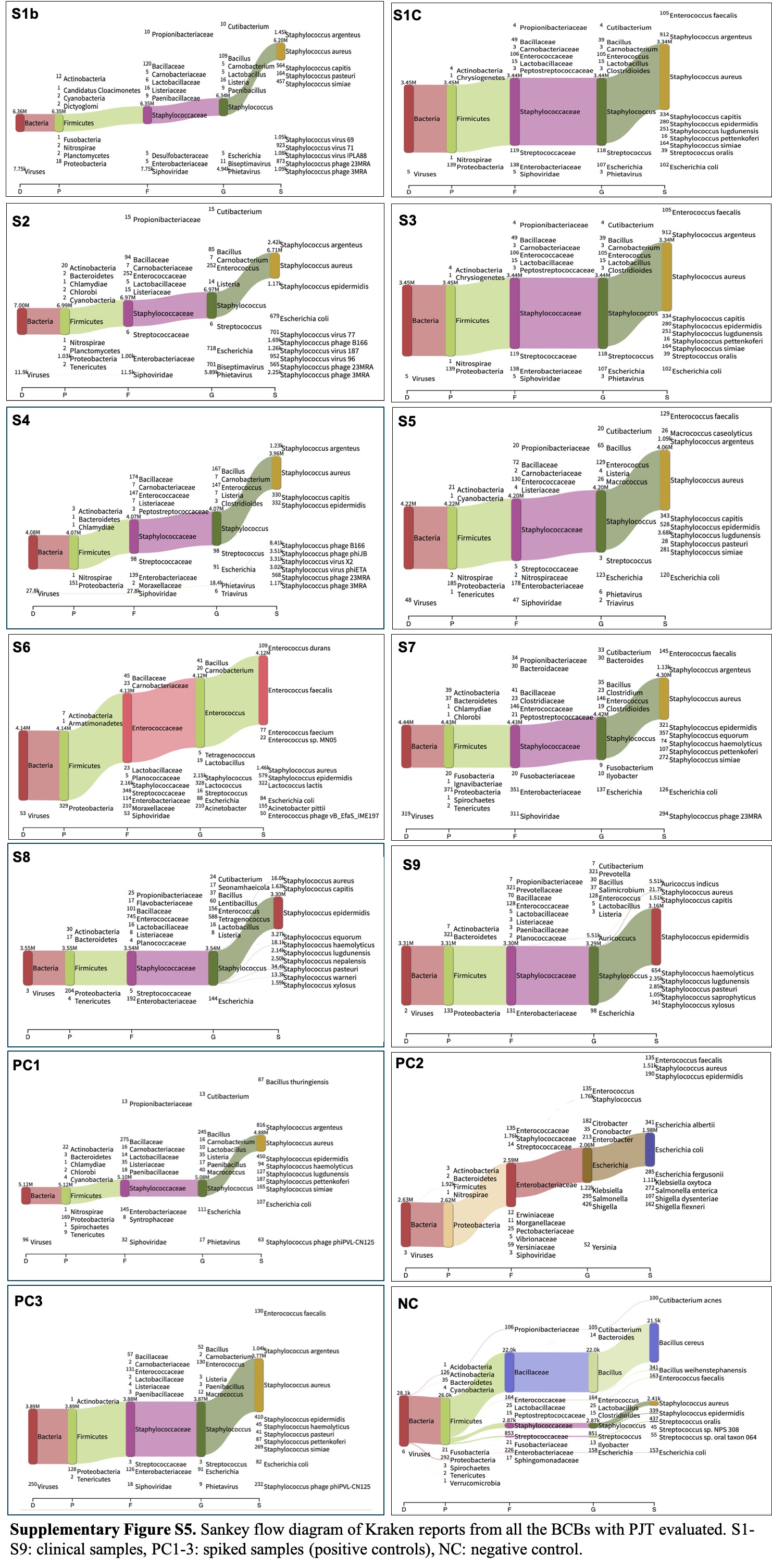

Supplement: Supplementary file 16 [file Image_5.JPEG]

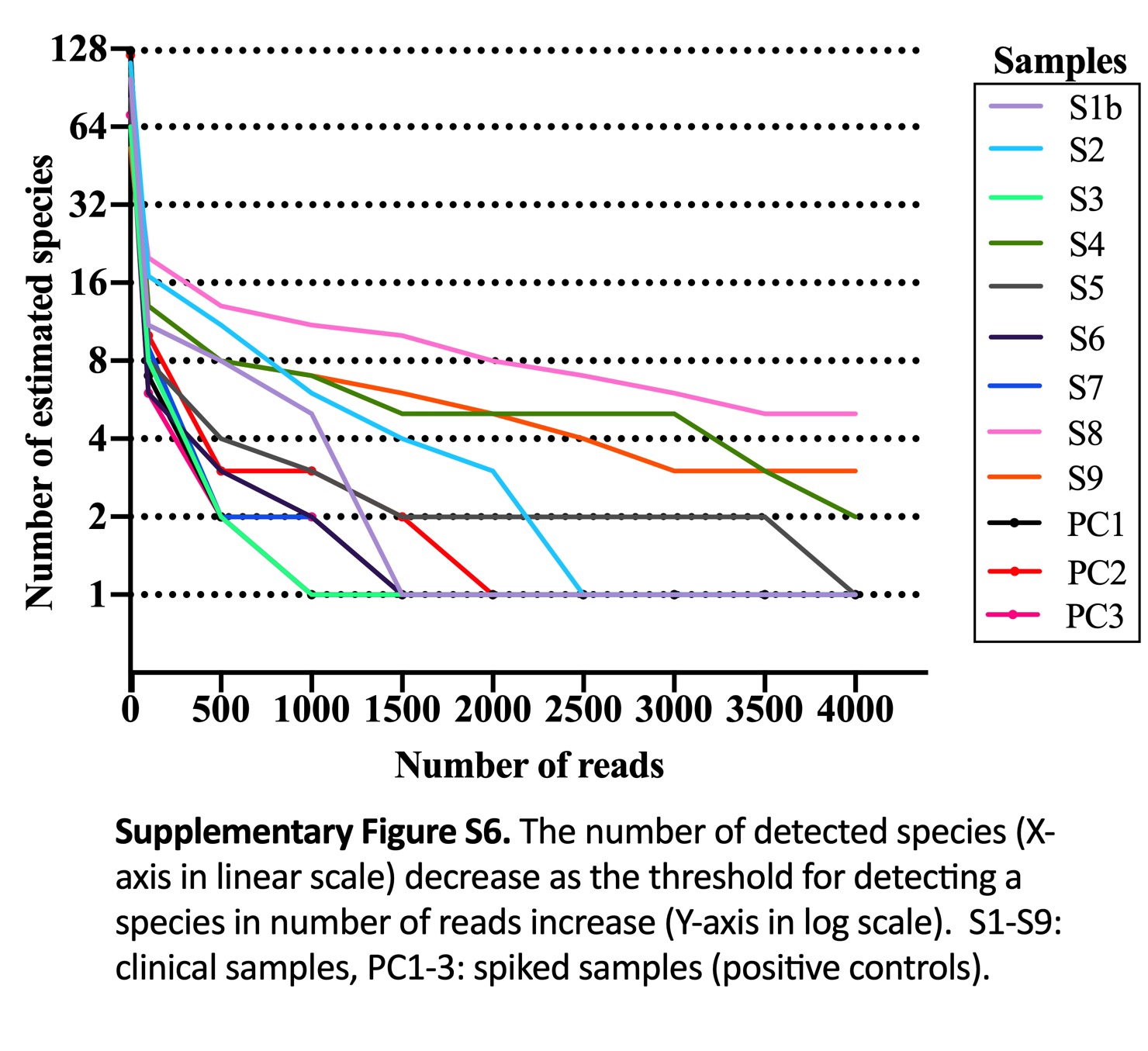

Supplement: Supplementary file 17 [file Image_6.JPEG]

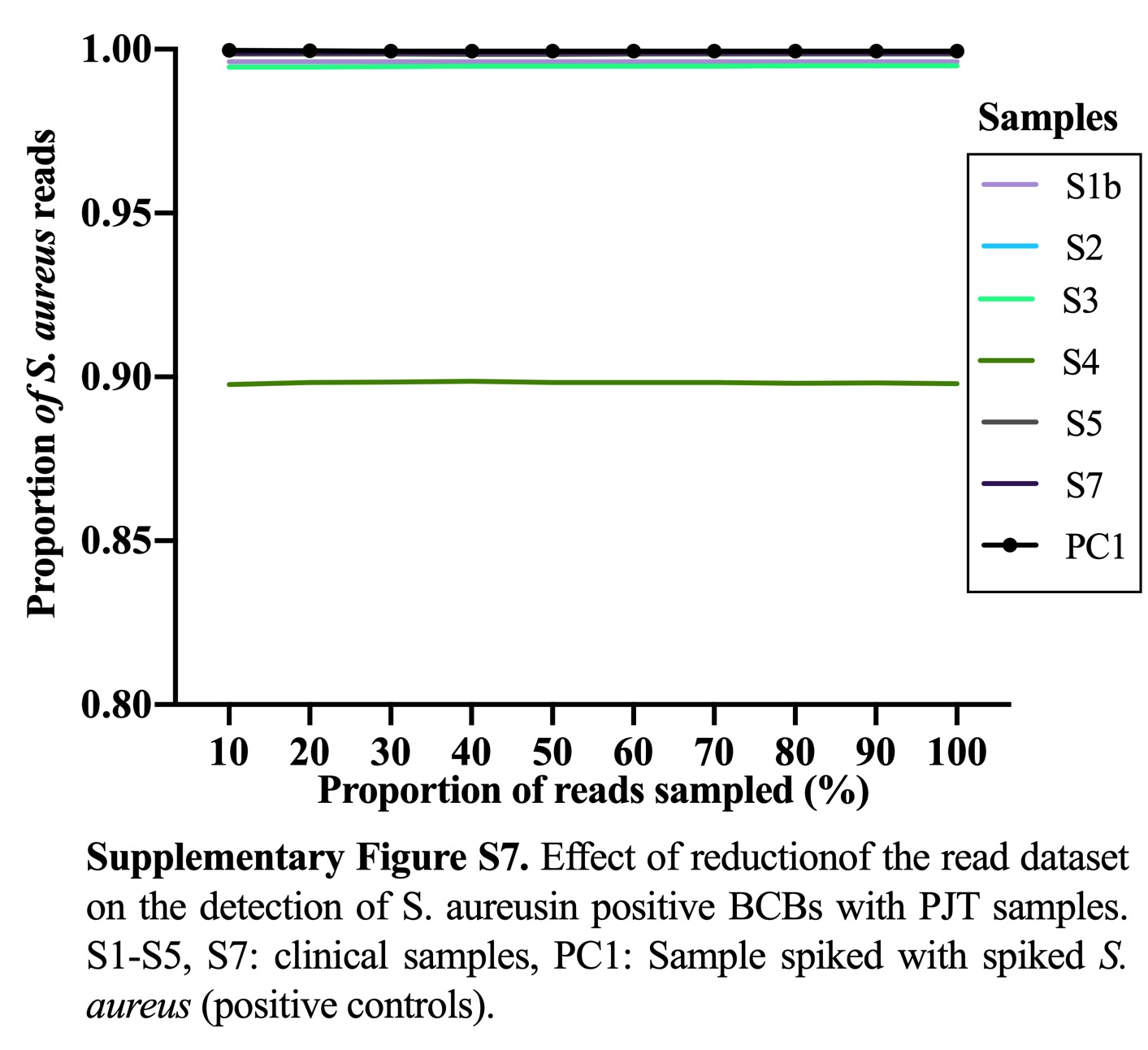

Supplement: Supplementary file 18 [file Image_7.JPEG]
